# Characterisation of *Kothea flammea* gen. nov., sp. nov., a planctomycete of the family *Pirellulaceae* isolated from Fjord Schlei in the Baltic Sea

**DOI:** 10.1038/s41598-026-52476-w

**Published:** 2026-05-20

**Authors:** Gaurav Kumar, Nicolai Kallscheuer, David Appiah, Jonathan Hammer, Tom Haufschild, Christian Jogler

**Affiliations:** 1 Department of Microbial Interactions, Institute of Microbiology, Friedrich Schiller University, Jena, Germany; 2 Cluster of Excellence Balance of the Microverse, Friedrich Schiller University, Jena, Germany

**Keywords:** *Planctomycetota*, Schlesner strain collection, *Roseimaritima* spp., *Candidatus* Laterigemmans baculatus, Brackish water, Genetics, Microbiology, Molecular biology

## Abstract

The flame-like reddish to orange-pigmented planctomycetal strain SH139^T^ was isolated from brackish water obtained from Fjord Schlei in Northern Germany as part of an isolation effort led by Heinz Schlesner at Kiel University between the 1980s and early 2000s. Single gene- and whole genome-based analyses employing five established phylogenetic markers indicate that the novel isolate constitutes a novel genus and species within the family *Pirellulaceae* in the phylum *Planctomycetota*. The strain is aerobic, heterotrophic, mesophilic, and exhibits neutrophilic to slight alkaliphilic properties. Its genome size and DNA G + C content of 7.8 Mb and 55.8%, respectively, are comparable to the current closest relatives. The results of the phenotypic and phylogenetic analyses are supported by comprehensive comparative genomic data, including pangenome reconstruction and profiling of carbohydrate-active enzymes and biosynthetic gene clusters. We thus introduce *Kothea flammea* gen. nov., sp. nov., that is constituted by the here characterized isolate SH139^T^ (= KCTC 102020^T^ = DSM 116129^T^) as type strain. The novel genus is named after Prof. Erika Kothe (Friedrich Schiller University Jena, Germany) in recognition of her pioneering work on microbial communication.

**Supplementary Information:**

The online version contains supplementary material available at 10.1038/s41598-026-52476-w.

## Introduction

At present, available axenic cultures encompass less than 1% of the suspected bacterial diversity^1^, while the remaining 99% of elusive microorganisms are classified as ‘microbial dark matter’^2^. Various cultivation strategies aiming at isolating fastidious bacterial strains in undersampled bacterial phyla have been developed^3^. However, the natural co-existence of yet untapped bacteria with complex communities of easily cultivable and rapidly growing strains often hinders enrichment and purification to yield axenic cultures of the former. Consequently, the targeted cultivation and deep-cultivation methodologies are essential for the isolation of novel, elusive microbes belonging to understudied bacterial phyla, such as *Planctomycetota*. Initially discovered almost a century ago, the first strain belonging to this phylum was misidentified as a floating fungus, leading to its name^4^. Strains of the phylum are ubiquitous and garner significant interest due to their distinctive cell biological characteristics. Prior research indicates that model strains within this phylum can import intact high molecular-weight polysaccharides, such as dextran, into an expanded periplasmic space, in which they are subsequently degraded into oligo- and monosaccharides^5^. This ‘selfish’ behavior, in contrast to the secretion of polysaccharide-degrading enzymes, may confer a competitive advantage in oligotrophic environments in which readily degradable sugars are scarce, and carbon must be derived from the breakdown of recalcitrant complex polysaccharides, such as those of plant or algal origin. Additional uncommon cellular characteristics include an enigmatic form of asymmetric cell division referred to as “budding” and the absence of otherwise essential bacterial cell division proteins including FtsZ, the hallmark protein for binary fission^6–8^. In silico genomic analyses of *Planctomycetota* strains highlight their potential to synthesize novel small molecules with prospective antimicrobial and health-promoting properties^9,10^. Recent investigations have further confirmed that metabolite extracts from this phylum exhibit antimicrobial activities^7,11–13^, positioning *Planctomycetota* as a promising, yet underexplored, reservoir of novel bioactive compounds. Till today, only a handful of compounds have been isolated from the phylum^14–19^.

The phylum *Planctomycetota* demonstrates remarkable metabolic versatility, facilitating the colonization of a broader range of ecological niches^7^ and the degradation of various polysaccharides^20,21^. Although predominantly characterized as aerobic, mesophilic, and heterotrophic, these bacteria can thrive over a spectrum of environmental conditions, such as pH, oxygen availability and temperature. Notably, members of the class “*Candidatus* Brocadiia” exhibit the unique ability to perform anaerobic ammonium oxidation (anammox)^22,23^. Strains belonging to the phylum have been documented in or isolated from a wide array of terrestrial habitats, including soils^24–26^ and peat bogs^27^, as well as from various aquatic environments, such as marine waters^28–33^, freshwater systems^26,34–36^, sediments^37,38^, non-natural plastic surfaces^39,40^ and deep-sea deposits^41^. *Planctomycetota* also exhibit adaptability to extreme environments, including hot springs^42^ and desert soils^43^. Numerous strains within this phylum have been isolated from biotic surfaces of potential symbiotic interaction partners, such as sponges^23,30,44–46^, macroalgae^47,48^, and also from the gastrointestinal tract^49^ or surface^50^ of other organisms, highlighting their ecological significance.

In this study, we report a novel isolate that phylogenetically affiliates with the family *Pirellulaceae* within the order *Pirellulales*. The order *Pirellulales*, belonging to the class *Planctomycetia*, is currently the best-characterized lineage within the phylum in terms of cultured representatives. With twenty described genera, *Pirellulaceae* represents the family with the highest number of described taxa in the entire phylum. Within the family, the current genus *Rosemaritima* comprises three species: *R. ulvae*, *R. sediminicola*, and *R. multifibrata*^29,48,51^. A closely related provisional genus, “*Candidatus* Laterigemmans baculatus”, is characterized on the basis of genomic data and exhibits a distinctive lateral budding mode of cell division^52^. The two genera are the closest neighbors of the here investigated novel isolate and are thus considered during comparison of analysed phenotypic and genomic features.

## Materials and methods

### Isolation, cultivation and initial identification of the strain

Strain SH139^T^ was originally isolated by Heinz Schlesner (Institute for Microbiology, Christian Albrechts University, Kiel, Germany) from surface water of Fjord Schlei, an estuary of the Baltic Sea close to Kiel in Northern Germany. The strain was originally isolated using M13(3x) medium and was re-inoculated from the cryogenic stock and routinely cultivated at 21 °C in the same medium. M13(3x) medium was composed of (per litre double distilled water; pH 7.8–8.0): peptone, 0.75 g; yeast extract, 0.75 g; artificial seawater (ASW), 250 mL (see below); Tris-HCl, 5 mL (final concentration of 5 mM); Hutner’s Basal Salts, 20 mL (see below). After autoclaving, the following sterile solutions were added: D-Glucose, 3 mL (of 250 g/L stock solution); vitamin solution, 5 mL (see below). The following supplemental solutions were prepared for the medium: 1 L vitamin solution contained *p*-aminobenzoic acid (0.01 g), biotin (0.004 g), pyridoxine hydrochloride (0.02 g), thiamine hydrochloride (0.01 g), sodium pantothenate (0.01 g), folic acid (0.004 g), riboflavin (0.01 g), nicotinamide (0.01 g) and vitamin B_12_ (0.0002 g). 1 L Mineral Salt Solution (Hutner’s Basal Salts) contained nitrilotriacetic acid (10 g), MgSO_4_ × 7 H_2_O (29.7 g), CaCl_2_ × 2 H_2_O (3.34 g), FeSO_4_ × 7 H_2_O (0.099 g) and Metal Salt Solution 44 (50 mL). 1 L of Metal Salt Solution 44 contained Na_2_-EDTA (0.25 g), ZnSO_4_ × 7 H_2_O (1.095 g), FeSO_4_ × 7 H_2_O (0.5 g), MnSO_4_ x H_2_O (0.154 g), CuSO_4_ × 5 H_2_O (0.0395 g), CoCl_2_ × 6 H_2_O (0.0203 g), and Na_2_B_4_O_7_ × 10 H_2_O (0.0177 g). 1 L ASW contained NaCl (24 g), MgCl_2_ × 6 H_2_O (5 g), Na_2_SO_4_ (4 g), CaCl_2_ × (1.1 g), KCl (0.7 g), NaHCO_3_ (0.2 g), KBr (0.1 g), H_3_BO_3_ (0.026 g), SrCl_2_ (0.024 g), and NaF (0.003 g). Solidified medium was prepared from 15 g/L agar in 200 mL double distilled H_2_O that was autoclaved separately and added prior to pouring of the plates. The 16S rRNA gene sequence of the isolate was amplified via polymerase chain reaction (PCR), purified using the NucleoSpin Gel and PCR Clean-up kit (Machery-Nagel), and subsequently sequenced at Macrogen Europe (Amsterdam, The Netherlands).

## Physiological analyses

The growth rate of strain SH139^T^ was assessed in three biological replicates with 5 mL culture each incubated in M13(3x) medium at 21 °C under shaking conditions (100 rpm). Optical density measurements at 600 nm (OD_600_) were recorded every 12 h for up to two weeks using a Biochrom UV-Vis spectrophotometer. To ascertain the optimal temperature for microbial growth, 100 µL of supernatant from an exponentially growing culture, devoid of visible aggregates, was spread on agar plates. These plates were incubated in duplicates at temperatures ranging from 4 °C to 42 °C (4, 10, 18, 21, 24, 28, 32, 37, 42 °C). Daily inspections were conducted, and growth was assessed by determining the time required for the formation of visible colonies or lawn. The temperature at which colonies or lawn appeared first was identified as the optimal growth temperature. The optimal pH for growth was determined in a 96-well cultivation experiment with continuous shaking over a cultivation time of two weeks. M13(3x) medium was supplemented with 100 mM of one of the following buffering agents: 2-(*N*-morpholino)ethanesulfonic acid (MES) for pH 5.0 and 6.0, 4-(2-hydroxyethyl)-1-piperazineethanesulfonic acid (HEPES) for pH 7.0, 7.5, and 8.0, or *N*-cyclohexyl-2-aminoethanesulfonic acid (CHES) for pH 9.0 and 10.0. Growth was quantified by measuring the OD_600_ in a BioTek Epoch2 microplate spectrophotometer (Agilent) at 21 °C. Each condition was tested in duplicates. Each measurement cycle lasted 30 min and was comprised of two shaking phases of 15 min interrupted by the OD_600_ measurements of the entire 96-well plate (Brand Plate pureGrade™ S, transparent sterile 96-well plates). The shaking regime of the 15 min interval was changed between linear to orbital to double orbital every 5 min. To mitigate condensation on the plate lid, a temperature gradient was established, maintaining the liquid at 21 °C and the lid temperature was maintained 2 °C above the cultivation temperature. For data analysis, the mean values of each time point were calculated, and the mean of the medium blank was subtracted. The growth rate at each pH was calculated from a selection of data points with the maximal slope of the natural logarithm of OD_600_ plotted against the cultivation time.

## Light microscopy and cell size determination

Light microscopy was performed following the methodology outlined in a previous study^53^. In brief, cells harvested from liquid cultures at the half-maximal OD_600_ were mounted on a 1% (w/v) agarose cushion prepared in deionized water (dH_2_O). Once the culture had dried on the agarose cushion, a coverslip was applied and secured at the edges with VLAP (a 1:1:1 mixture of vaseline, lanolin, and paraffin by weight) to ensure stability. Imaging was conducted using an inverted Nikon Ti2 microscope equipped with a Nikon Plan Apo λ 100x immersion oil objective, configured with a phase ring for phase-contrast (PhC) imaging or without for differential interference contrast (DIC) imaging^3^. The system included a Nikon DS-Ri2 camera and NIS-Elements software (version 5.30). Three-channel RGB images were processed in FIJI^54^ to generate single-channel RGB images. TIFF files were subsequently analyzed in BacStalk^55^, with cell segmentation performed using thresholds of 25 pixels for cell size and 15 pixels for minimum cell size. A total of three biological replicates, each comprising 150 cells, were evaluated. For data visualization, results were uploaded to SuperPlotsOfData^56^. To enhance visualization, brightness and contrast adjustments were manually applied to PhC and DIC images.

## Genomic DNA isolation, genome sequencing, annotation and analysis

Genomic DNA extraction, sequencing, assembly, and polishing were conducted following established protocols^53^. *De novo* genome assembly was performed using long-read data from Oxford Nanopore sequencing, with subsequent polishing utilizing short-read data from Illumina sequencing. For this, the sequencing reads were uploaded to the Galaxy web platform and the server available under the public domain usegalaxy.eu was used for the processing of the data using a customized workflow^57^. Details on the sequencing chemistry and bioinformatic workflow as well as tools, tool versions and optional parameters are provided in the Supplementary Materials in Table S1. Illumina sequencing was performed by Eurofins Genomics (Ebersberg, Germany). The genome completeness was assessed with BUSCO (version 5.8.2), while coding density and DNA G + C content were evaluated using CheckM (version 1.2.3). Following initial annotation with Prokka (version 1.14.5), the chromosome was re-oriented to the start codon of the *dnaA* gene, encoding the replication initiator protein, and subjected to final re-annotation using PGAP (version 2025-05-06, build 7983).

## Nucleotide sequence accession numbers

The 16S rRNA gene sequence of strain SH139^T^ has been deposited in the GenBank database under accession number PV955757. The genome sequence is available from NCBI under the accession number CP197414.

### Phylogenetic and genome-based analyses

The full-length 16S rRNA gene sequence of the novel isolate was retrieved from the genome annotated with Prokka and employed to identify the closest relatives via NCBI BLAST. Maximum likelihood phylogenetic trees were constructed based on 16S rRNA gene sequences and multi-locus sequence analysis (MLSA) for the novel strain and type strains of all species in the phylum *Planctomycetota*. The 16S rRNA gene sequences of the type strains of *Opitutus terrae* (NCBI accession no. AJ229235), *Kiritimatiella glycovorans* (accession no. NR_146840), and *Lentisphaera araneosa* (accession no. NR_027571), representing strains of the *Planctomycetota-Verrucomicrobiota-Chlamydiota* (PVC) superphylum outside of the phylum *Planctomycetota*, were used as outgroup in the 16S rRNA gene sequence-based tree. The sequence alignment was conducted using ClustalW^58^, and the phylogenetic tree were reconstructed with FastTree v2.2^59^ employing 1000 bootstrap replicates. The MLSA-based phylogeny was inferred using the autoMLST tool^60^ with 500 bootstrap replicates, including the genomes of *Planctopirus limnophila* DSM 3776^T^ (GenBank acc. no. CP001744.1), *Gimesia maris* DSM 8797^T^ (GenBank acc. no. CP042910.1) and *Rubinisphaera brasiliensis* DSM 5305^T^ (GenBank acc. no. CP002546.1), all from the family *Planctomycetaceae*, as outgroup. Phylogenetic trees were visualized using iTOL v6^61^. A 16S rRNA gene sequence similarity matrix was generated using TaxonDC^62^ based on the ClustalW alignment used for phylogenetic tree construction. Average amino acid identities (AAI) and average nucleotide identities (ANI) were calculated using scripts from the enveomics collection^63^. Additional phylogenetic markers, including sequence similarity of a 1298 bp partial sequence of the *rpoB* gene and percentage of conserved proteins (POCP), were determined following established methods^64,65^. The pangenome of selected strains was constructed using anvi’o v.8 with default parameters^66^. The same tool was also used for to assign the most closely related isolate or metagenome-assembled genome according to the Genome Taxonomy Database (GTDB). Biosynthetic gene clusters (BGCs) were predicted with antiSMASH v.8.0^67^ in relaxed mode with all extra features activated, and carbohydrate-active enzymes (CAZymes) were identified using dbCAN3^68^. The genomes of *Roseimaritima* spp. and “*Ca*. Laterigemmans sp.” were analysed in the same manner to ensure comparability of the data.

## Results and discussion

**Fig. 1:**
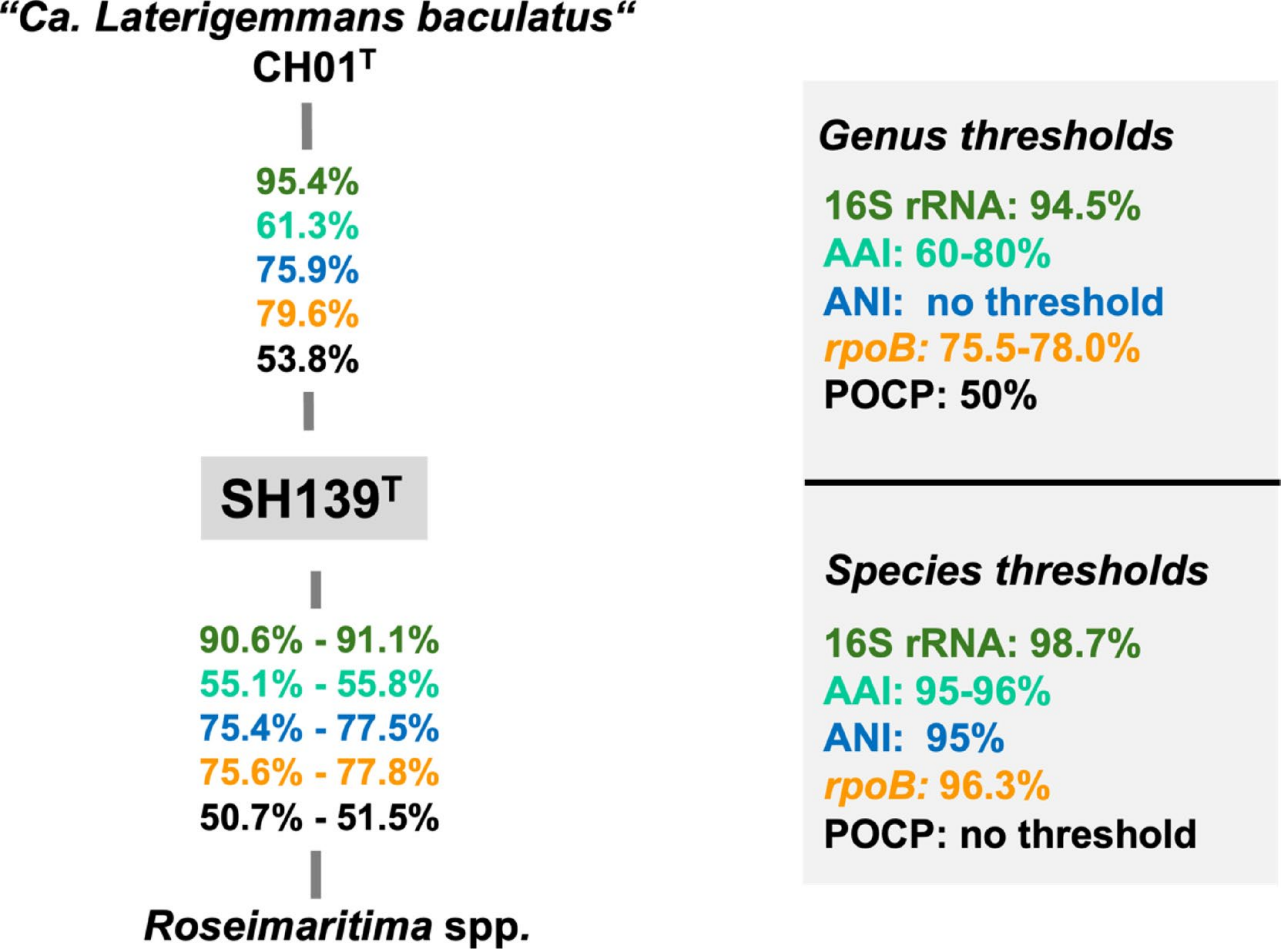
Comparison of phylogenetic markers for genus and species delineation. Markers used: 16S rRNA gene sequence identity (16S rRNA), average amino acid identity (AAI), average nucleotide identity (ANI), sequence similarity of a partial sequence of the *rpoB *gene (*rpoB*), percentage of conserved proteins (POCP).

**Fig. 2:**
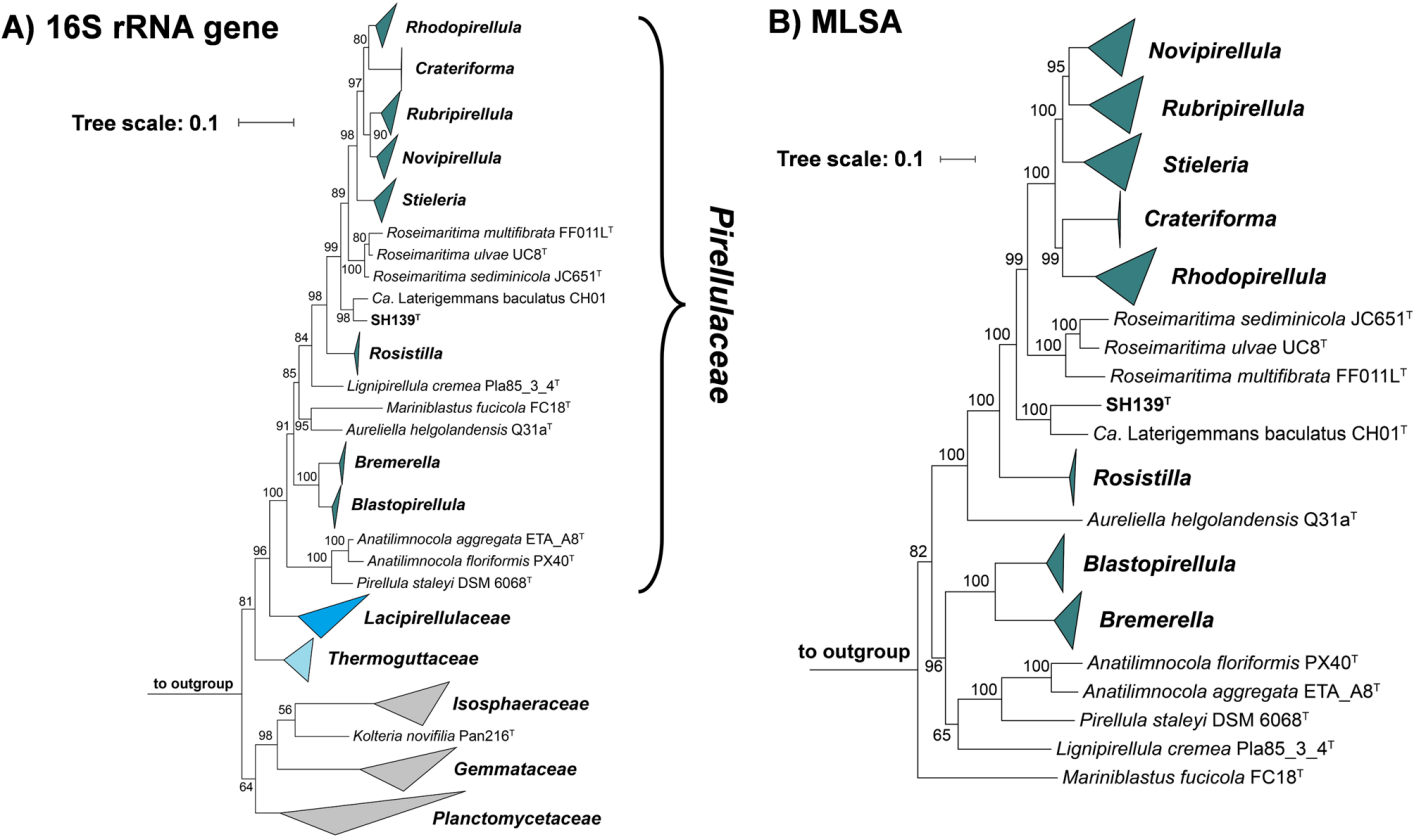
Phylogenetic placement. (**A**) Maximum likelihood phylogenetic tree based on 16S rRNA gene sequences showing the phylogenetic relationship of the novel isolate within the family *Pirellulaceae*. Bar, 0.1 substitutions per nucleotide position. (**B**) Multi-locus sequence analysis (MLSA)-based phylogenetic tree constructed with the genomes of characterized members in the family *Pirellulaceae*. The tree was computed based on a set of at least 30 single-copy gene-encoding proteins in a maximum likelihood approach with 500 bootstrap replications. Bar, 0.1 substitution per amino acid position. Bootstrap values for both trees are given at the nodes (in %). Phylogenetic trees were visualized with iTOL v6.

Initial nucleotide BLAST analyses of the 16S rRNA gene sequence of strain SH139^T^ against 16S rRNA gene sequences of cultivated strains resulted in a maximal similarity of 95.4% with “*Ca.* Laterigemmans baculatus” CH01^T^ and 91.1% with *Roseimaritima* spp. The similarity to “*Ca.* Laterigemmans baculatus” CH01^T^ slightly exceeds the 94.5% threshold typically used to define a new genus, but it is significantly below this threshold when compared with *Roseimaritima* spp.^65^(Fig. 1). The provisional taxon “*Ca.* Laterigemmans baculatus” retains its candidatus designation because the type strain failed to survive beyond a few subcultures, and it was not successfully deposited in any culture collection. Phylogenetic analyses and the clustering pattern in both phylogenetic trees (Fig. 2), corroborated the low sequence similarity, indicating a genus-level relationship with *Roseimaritima* spp., but showed species-level relationship with “*Ca.* Laterigemmans baculatus” CH01^T^. Furthermore, comparisons of AAI and *rpoB* values between strain SH139^T^ and *Roseimaritima* spp. revealed maximum similarities of 55.8% and 77.8%, respectively. These values fall below the recognized thresholds of 60–80% for AAI and in range of 75.5–78.0% for *rpoB* used for genus delineation^64,65,69^(Fig. 1). The comparison of POCP values between strain SH139^T^ and *Roseimaritima* spp. revealed maximum similarities of 51.5%, which is slightly above the genus threshold value of 50%. In summary, three out of four phylogenetic markers applicable for the delineation of genera support the conclusion of the introduction of a novel genus in case that this is also supported by differences in phenotypic and genomic characteristics of strain SH139^T^. In the past several novel genera were reported, although they had POCP values well above the threshold of 50% when compared with their closest described relatives^70^. NCBI BLAST analysis of the 16S rRNA gene sequence of the novel isolate (query coverage > 90%, sequence identity > 94.5%) and (meta)genomes of close relatives listed in the GTDB revealed that uncultured close relatives have been detected in a wide variety of environments, including microbial mats from hot water lakes, hypersaline mats, and wastewater treatment plants (Table S2). These results suggest that additional strains belonging to the proposed genus are likely to be cultured from diverse environmental sources in the future.

## Genomic characteristics

The genome size of the novel isolate SH139^T^ is 7.84 Mbp, which aligns with the range of genome sizes reported for *Roseimaritima* spp. that vary between 6.25 and 8.21 Mbp. Conversely, with 5.76 Mbp “*Ca.* Laterigemmans baculatus” CH01^T^ possesses the smallest genome within this group (Table 1). Despite phylogenetic markers indicating a species-level relationship between SH139^T^ and “*Candidatus* Laterigemmans baculatus” CH01^T^, the significant difference in their genome sizes suggests potential divergence. The DNA G + C content of strain SH139^T^ (55.8%) lies within the range observed for *Roseimaritima* spp. genomes (54.5–62.4%). Consistent with other species of the genus *Roseimaritima*, the novel isolate SH139^T^ is devoid of extrachromosomal elements. Its genome harbours ca. 724 protein-coding genes per Mbp, includes 1,391 genes encoding hypothetical proteins, and possesses the highest coding density (89%) and highest number of tRNA genes (96) among the five closely related strains. Like *R. multifibrata* FF011L^T^, strain SH139^T^ possesses duplicate copies of the 5S, 16S, and 23S ribosomal RNA genes, while the other *Roseimaritima* spp. possess single copies.

**Table 1:** Comparison of genomic and genome-encoded features of strain SH139^T^ to the closest relatives “*Ca*. Laterigemmans baculatus” CH01^T^, *Roseimaritima ulvae* UC8^T^, *Roseimaritima sediminicola* JC651^T^, and *Roseimaritima multifibrata* FF011L^T^.

Characteristics	SH139^T^	“*Ca*. Laterigemmans baculatus” CH01^T^	*R*. *sediminicola* JC651^T^	*R*. *ulvae* UC8^T^	*R*. *multifibrata* FF011L^T^
*Genomic features*					
Genome size (bp)	7,837,734	5,758,428	6,245,843	8,212,515	7,216,590
Contigs	1	150	185	1	1
Plasmids	no	inconclusive	inconclusive	no	no
DNA G + C (%)	55.8	62.4	62.4	59.1	54.5
Genes	5840	4288	4599	5829	5248
Genes/Mbp	745	745	736	710	727
Protein-coding genes	5675	4193	4504	5727	5167
Protein-coding genes/Mbp	724	728	721	697	716
Hypothetical proteins*	1391	876	956	1283	1130
Hypothetical proteins (%)	24.5	20.9	21.2	22.4	21.9
Coding density (%)	89.0	86.7	86.5	87.5	85.4
rRNA genes (5S,16S,23S)	2,2,2	2,2,1	1,1,1	1,1,1	2,2,2
tRNA genes	96	59	46	67	43
*Secondary metabolite-associated biosynthetic gene clusters*					
Terpene biosynthesis	4	3	4	3	3
Type I polyketide synthase	2	1	1	4	1
Type III polyketide synthase	1	1	1	1	1
Non-ribosomal peptide synthetase (NRPS)	2	0	0	1	2
NRPS-like protein	3	2	1	2	2
Ribosomally synthesised and post-translationally modified peptide product (RiPP)	0	0	0	0	1
RiPP-Like	0	0	0	0	1
Heterocyst glycolipid synthase-like PKS (hglE-KS)	1	0	0	0	0
Acyl amino acids	1	1	0	1	0
*Carbohydrate-active enzymes*					
Glycoside hydrolases	47	42	42	60	47
Glycosyltransferases	74	52	53	76	64
Polysaccharide lyases	2	4	1	5	9
Carbohydrate esterases	22	21	21	25	31
Carbohydrate-bind. modules	18	10	16	23	15
Auxiliary activities	2	5	2	4	1
CAZyme genes (total)	165	134	135	193	167
CAZyme genes per Mbp	21	23	22	24	23

*based on the Refseq/PGAP-annotated genomes

### Pangenome reconstruction and evaluation of genome-encoded functions

**Fig. 3:**
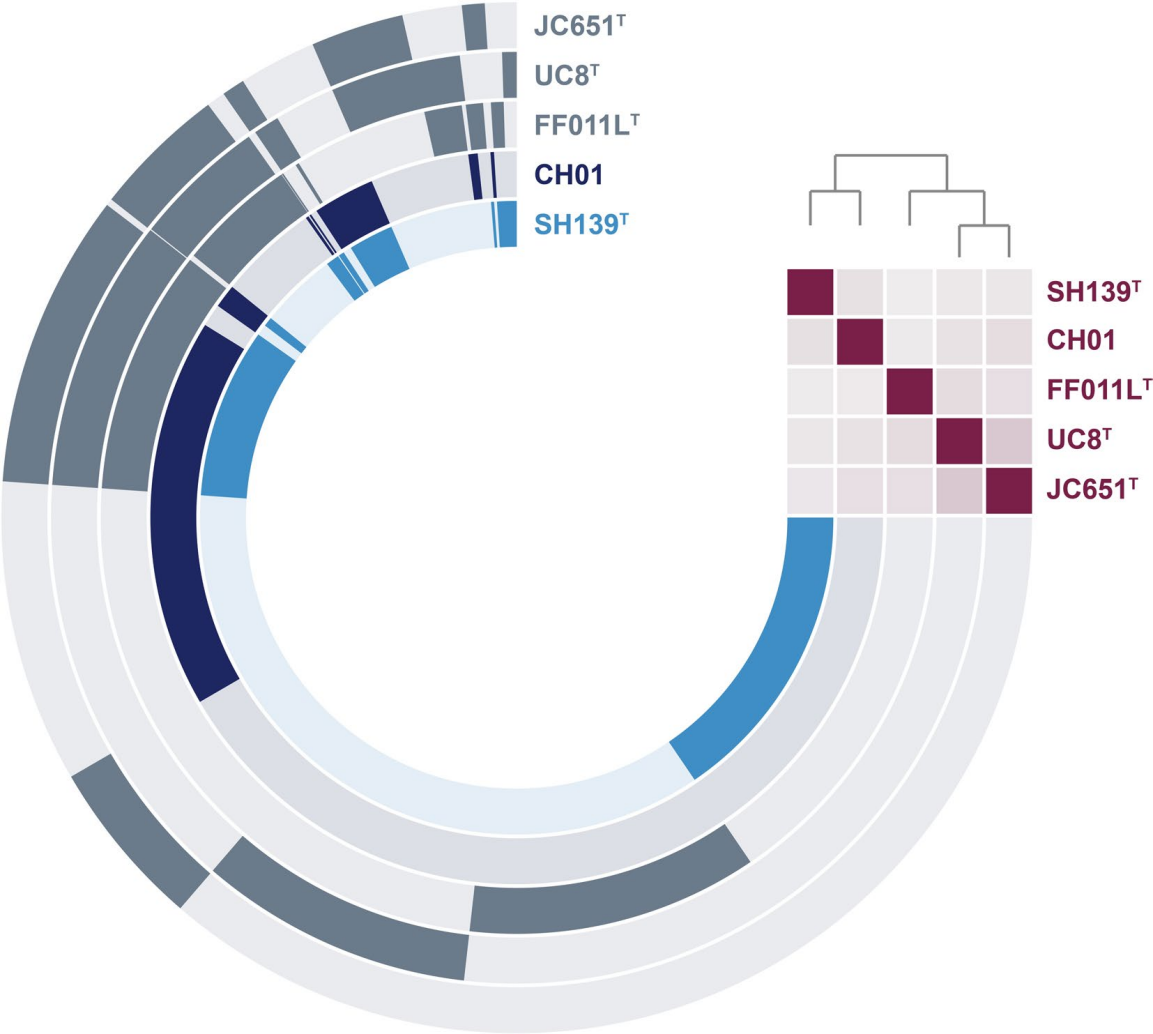
Pangenome reconstruction. Each open circle represents the pangenome of all strains but is colored darker when the gene is present in the respective genome. The heatmap in the upper right corner indicates the degree of relationship based on ANI values (ANI ≤ 70%, pale burgundy red to ANI = 100%, bright burgundy red).

To visualize the genome-based similarity among the compared strains, a pangenome was constructed and analyzed. This pangenome, encompassing the genomes of all five compared species including strain SH139^T^, comprises 14,234 clusters, of which 1,459 are conserved across all five strains (core genome). The remaining clusters are either unique to individual strains (singletons) or not conserved across all strains. The singleton gene count for strain SH139^T^ is 2,943. Within the pangenome visualization, beyond the core genome shown at the 9–10 o’clock segment, strain SH139^T^ and “*Ca.* Laterigemmans baculatus” CH01^T^ exhibit shared accessory genes, as evidenced at the 11 o’clock position. Correspondingly, each of the three *Roseimaritima* species possesses distinct supplementary genes, similarly represented at the 11 o’clock position in the pangenome visualization (Fig. 3). These findings reflect the species-level affiliation between the novel isolate SH139^T^ and “*Ca.* Laterigemmans baculatus” CH01^T^, while confirming a more divergent evolutionary relationship with the three *Roseimaritima* species.

Genome mining for secondary metabolite-associated biosynthetic gene clusters (BGCs) using antiSMASH identified 14 BGCs in the genome of strain SH139^T^ (Table 1). Four of these BGCs are likely involved in the biosynthesis of terpenoids or their precursors. Other predicted BGCs include genes encoding putative type I or type III polyketide synthases (PKSs) or are linked to the synthesis of short peptides formed by non-ribosomal peptide synthetase (NRPS)-like enzymes or heterocyst glycolipid synthase-like PKSs (hglE-KS). Across all five compared strains, the identification of CAZyme-encoding genes resulted in 134–193 hits per genome, with comparable numbers of hits in the classes of glycoside hydrolases and glycosyltransferases, though these numbers were slightly higher in *R. ulvae* UC8^T^ (Table 1). This is not surprising since the genome of *R. ulvae* UC8^T^ is 0.5 Mbp larger than that of the strain SH139^T^. The counts of genes encoding polysaccharide lyases or proteins with auxiliary activities fall below ten per genome for all five genomes analyzed. This indicates that all five strains can in principle degrade complex carbohydrates into simpler sugars, however, the exact substrate spectrum needs to be determined in future cultivation experiments based on the axenic cultures and cannot be reliably predicted from the genome sequence. The genome of strain SH139^T^ indicates its ability to produce cyanophycinase enzymes, which are essential for breaking down cyanophycin, a compound of biotechnological interest as a source of polyaspartic acid. The genome also encodes multiple genes potentially conferring resistance to heavy metals, including cobalt, zinc, and cadmium. This may further underscore the environmental importance of the novel planctomycetal isolate.

### Physiological and phenotypic characterization

**Fig. 4:**
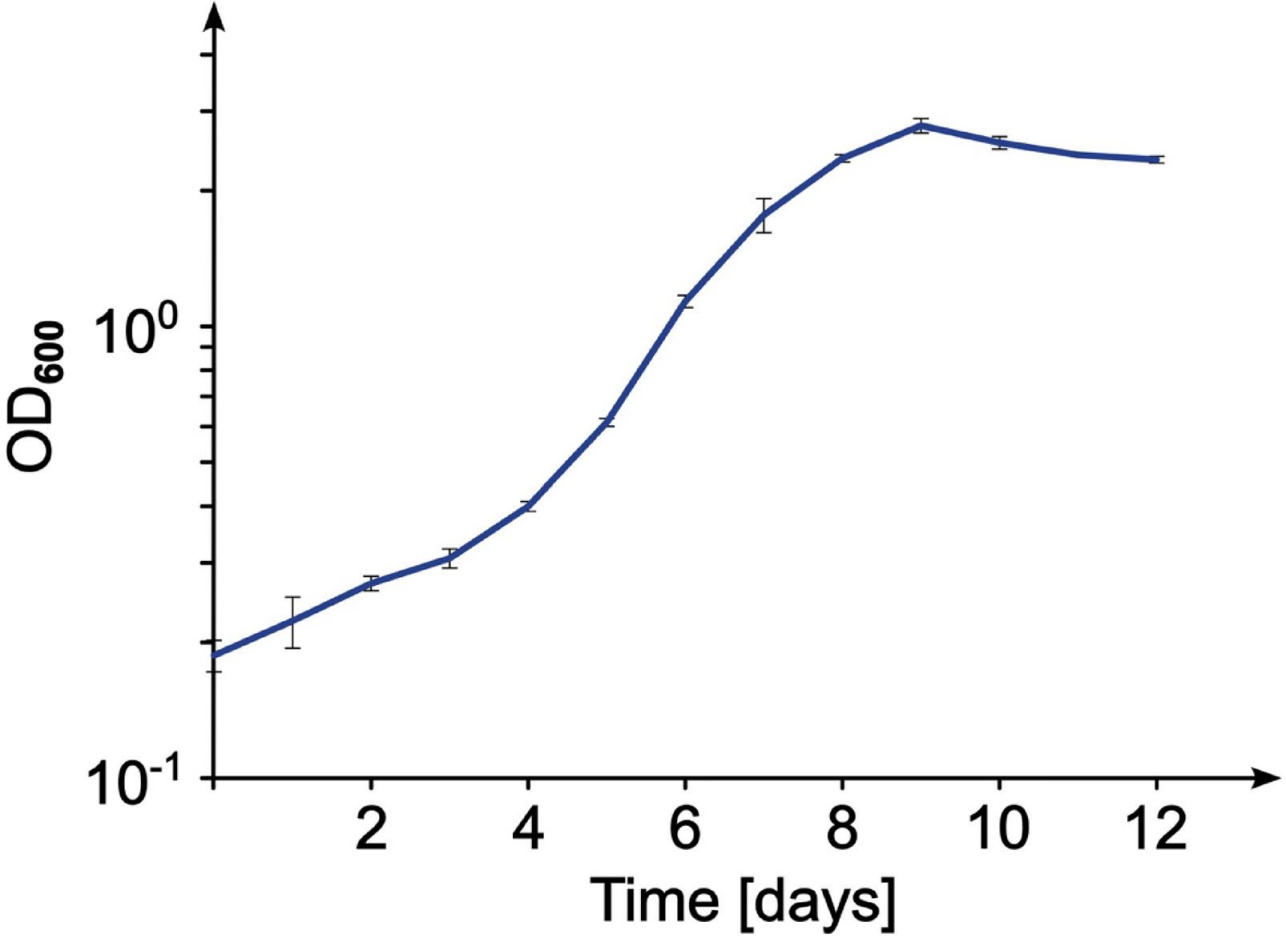
Growth curve. The graph shows a typical growth curve of strain SH139^T^. Data points at each time represent the average of three biological replicates. Doubling times were derived from optical density (OD_600_) measurements in the exponential phase. The error bars indicate the standard deviation.

**Fig. 5:**
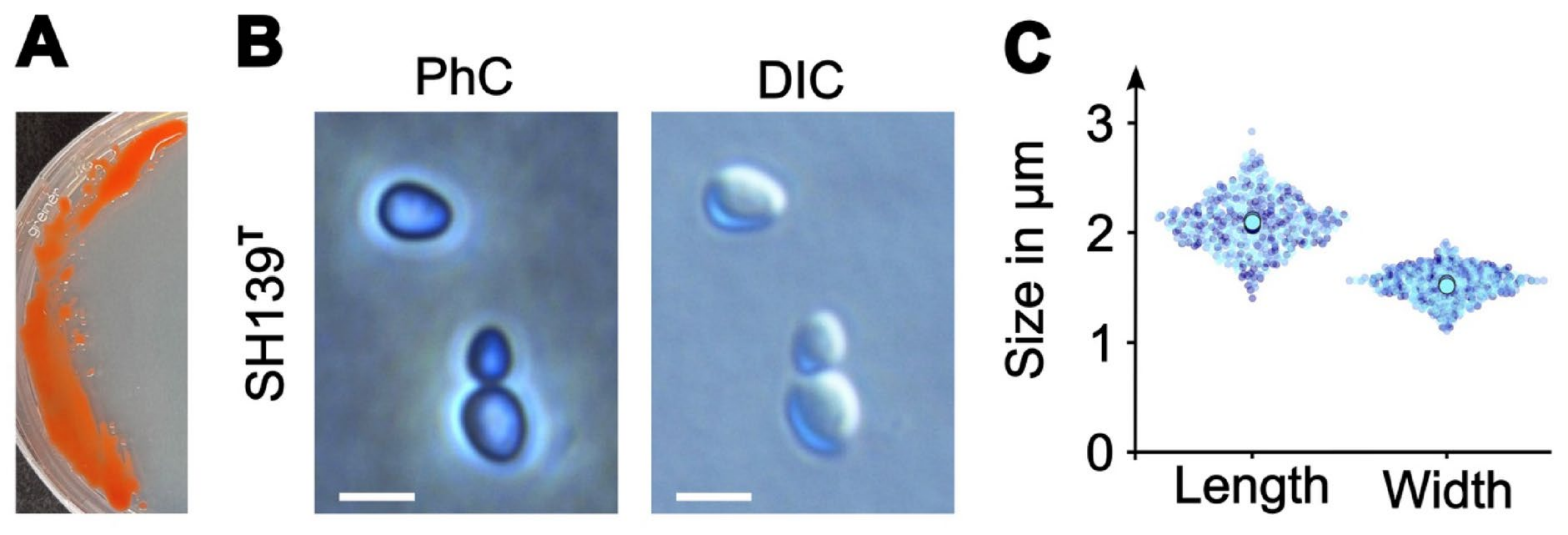
Colony appearance, cell morphology and cell size of strain SH139^T^. Appearance of colonies on plates, and cell morphology of strain SH139^T^ using phase contrast (PhC) and differential interference contrast (DIC) were examined. On agar plates, colonies appear big, irregular and have a slimy consistency (**A**). An individual cell and a cell during cell division are depicted with a larger mother and smaller daughter cell still connected to each other (**B**). Cell size of strain SH139^T^ obtained from three replicates (visualized by different shades of blue), mean values of each replicate are depicted by larger circles (**C**). Scale bars represent 2 μm.

Strain SH139^T^ displays a maximal growth rate of 0.017 h⁻¹ which corresponds to a doubling time of ca. 41 h (Fig. 4). Hence, the novel strain grows relatively slowly compared to most of the characterized planctomycetal isolates (typical growth rates range from 0.01 to 0.07 h^− 1^)^71,72^. In both, agar plate and liquid cultures, the novel strain displays an intense flame-like reddish-orange pigmentation, different from that of its closest relatives, “*Ca.* Laterigemmans baculatus” CH01^T^, and *Roseimaritima* spp. Colonies of the novel strain show either circular or irregular shapes with a more mucoid texture (Fig. 5A). Strain SH139^T^ grows at temperatures between 18 and 28 °C, with optimal growth at 21 °C, and tolerates a narrow pH range of 7.0–8.0, with an optimum at pH 7.5 (Table 2). Like its closest relatives, it also exhibits an aerobic and heterotrophic lifestyle. On the microscopic level, cells appear pear-shaped and have a mean cell length and width of 2.1 ± 0.2 μm and 1.5 ± 0.1 μm, respectively (Fig. 5B, C). This observation renders the cells of strain SH139^T^ smaller than those of the closest relative “*Ca.* Laterigemmans baculatus” CH01^T^, which have a length of 3.0 to 4.0 μm and a width of 0.5 to 0.8 µm^52^. Strain CH01^T^ was also observed to divide via lateral budding, a form of planctomycetotal cell division in which the daughter cell is formed on the lateral side instead of the pole of the mother cell^52^. Along with *Kolteria novifilia* Pan216^T^, *Alienimonas chondri* LzC2^T^ and some others^7,73,74^, strain CH01^T^ thus shows variations from bud formation exactly at the cell pole. In contrast to this observation, we could observe cells of strain SH139^T^ to divide via polar cell division (polar budding), the common form of cell division in the class *Planctomycetia* as also observed for the genus *Roseimaritima*. Here, the daughter cell emerges on one of the cellular poles, elongates (Fig. 5B) and subsequently pinches off from the mother cell to start a new cell cycle.

**Table 2:** Comparison of phenotypic characteristics of strain SH139^T^ to the closest relatives “*Ca*. Laterigemmans baculatus” CH01^T^, *Roseimaritima ulvae* UC8^T^, *Roseimaritima* *sediminicola* JC651^T^, and *Roseimaritima multifibrata* FF011L^T^.

Characteristics	SH139^T^	“*Ca*. Laterigemmans baculatus” CH01^T^	*R*. *sediminicola* JC651^T^	*R*. *ulvae* UC8^T^	*R*. *multifibrata* FF011L^T^
*Sampling Information*					
Location	Kiel, Germany	Odisha, India	Odisha, India	Northern coast, Portugal	Helgoland, Germany
Sampled Material	surface water of Fjord Schlei, estuary of the Baltic Sea	sediment, Chilika lagoon	sediment, Chilika lagoon	marine macroalga (*Ulva* sp.)	marine macroalga (*Laminaria* sp.)
*Phenotypic features*					
Pigmentation	reddish-orange	dark pink	light pink	light pink	pink to red
Cell Shape	pear-shaped	rod-shaped	round to pear-shaped	spherical to ovoid	elongated pear-shape
Size (Length x width) (µm)	2.1 ± 0.2 x 1.5 ± 0.1	3.0–4.0 x 0.5–0.8	1.0–2.0 x 0.8–1.5	1.1–1.8 x 0.9–1.5	2.0 ± 0.3 x 1.0 ± 0.2
Cell Division Mode	asymmetric (polar budding)	asymmetric (lateral budding)	asymmetric (polar budding)	asymmetric (polar budding)	asymmetric (polar budding)
Temperature range (optimum) (°C)	18–28 (21)	n.d.	15–40 (25)	15–35 (30)	12–30 (26)
pH range (optimum)	7.0–8.0 (7.5)	n.d.	6.0–9.0 (7.5)	6.0–10.0 (7.5)	5.5–10.0 (7.5)
Relation to Oxygen	aerobic	aerobic	aerobic	aerobic	aerobic
Aggregates	yes	yes	yes, rosettes	yes, rosettes	yes, rosettes

n.d., not determined. Features of *“Ca.* Laterigemmans baculatus” CH01^T^, *R. ulvae* UC8^T^, *R. sediminicola* JC651^T^, and *R. multifibrata* FF011L^T^ were obtained from the respective species descriptions.

## Conclusion

Based on the analysis of phylogenetic markers and supported by phenotypic and genomic differences, we conclude that the analyzed isolate belongs to a novel genus and species of the family *Pirellulaceae*. Thus, we propose the name *Kothea flammea* gen. nov., sp. nov., with the novel species being represented by SH139^T^ as the type strain. Since strain SH139^T^ does not divide by lateral budding, it was decided to assign it to a separate genus and not to assign it to the provisional genus “*Ca*. Laterigemmans”.

### Description of *Kothea *gen. nov.

Ko.the’a. N.L. fem. n. *Kothea*, named in honor of Prof. Dr. Erika Kothe, Friedrich Schiller University Jena, for her outstanding contributions in microbial communication.

Strains belonging to the genus are heterotrophic, aerobic, mesophilic and neutrophilic. Cells divide by polar budding. Laterally budding cells were not observed. The DNA G + C content is around 56%. The genus belongs to the family *Pirellulaceae*, order *Pirellulales*, class *Planctomycetia*, phylum *Planctomycetota*. The type species of the genus is *Kothea flammea.*

### Description of *Kothea flammea *sp. nov.

L. fem. adj. *flammea*, flame-colored, fiery red, referring to the reddish to orange pigmentation of the type strain.

Cells produce flame-like reddish-orange pigmented colonies and are pear-shaped, measuring approximately 2.1 × 1.5 μm. The type strain is SH139^T^ (= KCTC 102020^T^ = DSM 116129^T^). It was isolated from Fjord Schlei, part of the Baltic Sea in Northern Germany. The type strain grows optimally at a pH of 7.5 (range 7.0–8.0) and a temperature of 21 °C (range 18–28 °C). The doubling time of the type strain in M13(3x) medium is around 41 h. The type strain genome is 7.84 Mb in size and has a DNA G + C content of 55.8%. The type strain lacks extrachromosomal elements.

## Supplementary Information

Below is the link to the electronic supplementary material.

Supplementary Material 1

Supplementary Material 2

## Data Availability

The 16S rRNA gene sequence of strain SH139^T^ has been deposited in the GenBank database under accession number PV955757. The genome sequence is available from NCBI under the accession number CP197414.
